# Collaborative Practice in Oral Nutritional Supplement Provision: The Critical Role of Pharmacists in the Patient Journey

**DOI:** 10.3390/healthcare14121673

**Published:** 2026-06-12

**Authors:** Željko Krznarić, Darija Vranešić Bender, Dina Ljubas Kelečić, Nikica Daraboš, Ivan Radoš, Ana Soldo

**Affiliations:** 1 Unit of Clinical Nutrition, Department of Internal Medicine, University Hospital Centre Zagreb, Kišpatićeva 12, 10000 Zagreb, Croatia; zeljko.krznaric60@gmail.com (Ž.K.); ljubas.dina@gmail.com (D.L.K.); 2 Department of Physiotherapy, University North, Ulica 104. Brigade 3, 42000 Varaždin, Croatia; darabos.dr@googlemail.com; 3 Medical Nutrition Industry Coordination—CEA, Radnička Cesta 37a, 10000 Zagreb, Croatia; ivanrados1986@gmail.com; 4 Croatian Chamber of Pharmacists, Martićeva Ulica 27, 10000 Zagreb, Croatia; ana.soldo@hljk.hr

**Keywords:** oral nutritional supplements (ONSs), disease-related malnutrition (DRM), collaborative practice, pharmacist role, patient adherence, healthcare communication, co-payment barriers, nutritional therapy

## Abstract

**Highlights:**

**What are the main findings?**

- While 92% of patients accepted prescribed oral nutritional supplements (ONSs), 51% of first-time users arrived at pharmacies uninformed about co-payment requirements, creating a critical information gap that reduced immediate acceptance from 93% (informed patients) to 33% (uninformed patients), *p* < 0.05.
- Multivariate analysis identified prior knowledge of co-payment as the strongest predictor of ONS acceptance (OR = 0.027, 95%CI: 0.003–0.250, *p* < 0.001), with informed patients showing 97% reduced odds of refusal, while patient characteristics (diagnosis, age, comorbidities) were not significant predictors.

**What are the implications of the main findings?**

- The 8–12% rate of non-acceptance or dose reduction at the pharmacy dispensing encounter was associated with unmet need for enhanced collaborative practice between prescribing physicians and pharmacists, with systematic communication protocols ensuring patients receive explicit co-payment information and therapeutic rationale during prescription encounters.
- Pharmacist education on disease-specific nutritional needs and ONS counseling skills may be relevant to address, as 15–22% of patients required pharmacist intervention before accepting their prescription, and pharmacists identified lack of ONS knowledge as their primary educational gap.

**Abstract:**

Background/Objectives: Disease-related malnutrition affects millions of patients worldwide. Nutrition support therapy (NST), namely oral nutritional supplements (ONSs), serve as a cornerstone therapeutic intervention. However, treatment effectiveness depends not only on an appropriate prescription but also on patient acceptance and adherence. This study evaluates the provision pathway of ONSs within a co-payment healthcare system, focusing on patient acceptance patterns, barriers to adherence, and the critical yet underexplored role of pharmacist–patient interactions in determining treatment outcomes. Methods: A cross-sectional observational study was conducted across 100 Croatian community pharmacies during September–October 2025. Pharmacists prospectively documented 973 patient encounters involving ONS prescriptions requiring co-payment using real-time patient record forms. Data captured patient demographics, diagnoses, prescription patterns, prior knowledge of co-payment requirements, acceptance responses, and pharmacist-assessed reasons for refusal. Results: While 65% of all patients knew about co-payment requirements in advance, 51% of first-time users arrived uninformed, leading to dramatically different acceptance patterns (93% immediate acceptance when informed versus 33% when uninformed, *p* < 0.05). Overall, 8–12% of patients refused or reduced prescribed ONSs. Among refusals, 59% cited the financial burden, but, critically, 23% appeared not to understand why an ONS was prescribed or what benefits to expect, revealing significant communication gaps in the care pathway. Overall, fifteen percent of patients required an explanation from the pharmacist before accepting their prescription, demonstrating pharmacists’ decisive role as gatekeepers of nutritional therapy. Conclusions: These findings suggest that the pharmacy dispensing encounter is an important decision point in the ONS care pathway, where insufficient preparation and coordination may be associated with suboptimal treatment outcomes among vulnerable patient populations. Improved prescriber–patient communication about co-payment and clinical rationale, pharmacist education in disease-specific nutrition and ONS counseling, and structured communication protocols between prescribers and pharmacists represent areas that may warrant further attention and evaluation.

## 1. Introduction

Disease-related malnutrition (DRM) represents a significant public health challenge affecting millions of patients worldwide, particularly those with chronic conditions, oncological diseases, and age-related decline. DRM in various clinical settings is associated with increased morbidity, mortality, prolonged hospital stays, rehospitalizations and reduced quality of life. The economic burden is substantial, with malnourished patients requiring more healthcare resources and experiencing poorer treatment outcomes [1,2].

Oral nutritional supplements (ONSs) serve as a cornerstone intervention for managing disease-related malnutrition. These specialized medical nutrition products provide energy, protein, essential nutrients and pharmaconutrients designed for patients who cannot meet their nutritional needs through regular diet alone, either temporarily or permanently [3]. In the European Union, ONSs are regulated under the category of Foods for Special Medical Purposes (FSMP), primarily governed by Regulation (EU) No. 609/2013 on foods for specific groups and further specified by Commission Delegated Regulation (EU) 2016/128 [4,5]. FSMP is defined as “food specially processed or formulated and intended for the dietary management of patients, including infants, to be used under medical supervision; it is intended for the exclusive or partial feeding of patients with a limited, impaired or disturbed capacity to take, digest, absorb, metabolize or excrete ordinary food or certain nutrients contained therein, or metabolites, or with other medically determined nutrient requirements, whose dietary management cannot be achieved by modification of the normal diet alone” [5].

Clinical evidence demonstrates that appropriate ONS use improves nutritional status, supports recovery, reduces complications, and enhances patient outcomes across various conditions including cancer, gastrointestinal diseases, chronic obstructive pulmonary disease (COPD), neurological diseases, geriatric medicine, and perioperative care [6,7,8,9]. However, the effectiveness of ONSs depends not only on appropriate clinical prescription but also on patient adherence and accessibility [10]. Reimbursement of ONSs by the national healthcare fund is shown to facilitate ONS consumption and increase adherence in many studies [10,11]. In healthcare systems where ONSs require patient co-payment, the step from prescription to actual use becomes a critical barrier to adherence [9,10]. It involves several stakeholders requiring coherent collaborative practice: specialists who diagnose malnutrition and prescribe treatment, clinical dietitians who advise on nutrition support therapy, general practitioners who coordinate care, pharmacists who dispense and advise, and patients who must understand and follow the therapy [3,12,13].

Despite this, the ONS provision pathway often operates as a fragmented sequence rather than a cohesive system [14], and the pharmacy dispensing encounter—the point at which prescribed therapy is first presented to the patient—remains particularly underexamined [15]. While specialists, clinical dietitians and general practitioners identify nutritional needs and prescribe treatment, they typically lack direct insight into what occurs at this encounter. Conversely, pharmacists interact directly with patients at the point of dispensing but often lack comprehensive clinical context about the prescribing rationale and patient-specific considerations [9]. Research indicates that 87.2% of pharmacists identify lack of expertise in nutrition counseling as a primary barrier to providing nutritional support, with limited educational resources further constraining their practice [15]. Although pharmacists are well positioned to identify barriers to ONS uptake—including affordability concerns, poor palatability, and treatment fatigue—their contribution to nutritional care remains underexplored in the literature [9,10,12].

This study examines the ONS provision pathway in Croatia’s co-payment healthcare system, focusing on patient acceptance patterns, barriers to adherence, and the role of pharmacist–patient interactions in determining treatment outcomes. Croatia provides an instructive case study as a European Union member state with a mainly public healthcare system where some categories of ONSs require substantial patient co-payment, creating financial barriers like those experienced in many healthcare systems globally.

The research investigates fundamental questions about the ONS care pathway:

1. What factors influence patients’ willingness to accept and collect prescribed ONSs at the pharmacy dispensing encounter?
2. What proportion of patients experience barriers—financial, informational, or psychological—that prevent them from obtaining prescribed nutrition therapy?
3. How do pharmacist–patient interactions influence acceptance decisions, and what role do pharmacists currently play in supporting nutritional therapy?
4. What gaps exist in the communication pathway from prescriber through pharmacist to patient, and how might enhanced collaborative practice optimize this crucial stage in the care pathway?

Identifying gaps at the pharmacy dispensing encounter may inform targeted interventions to strengthen collaborative practice across prescribers, pharmacists, and patients.

## 2. Materials and Methods

### 2.1. Study Design and Rationale

This study employed a cross-sectional design conducted in Croatian community pharmacies between September and October 2025. Data were collected prospectively using structured patient record forms (PRFs), completed by pharmacists immediately following patient encounters involving oral nutritional supplement (ONS) prescriptions requiring co-payment. This approach captures observed patient behavior at the point of dispensing and minimizes recall bias through immediate documentation. However, several variables—including patients’ prior knowledge of co-payment requirements, reasons for refusal, and patient understanding of therapeutic rationale—were recorded based on the pharmacist’s assessment of the interaction rather than direct patient self-report. These variables should therefore be understood as pharmacist-assessed proxies rather than validated patient-reported outcomes and may be subject to observer and interpretation bias.

### 2.2. Sample Design and Recruitment

Pharmacist Sample: The sample was designed as a structured pragmatic sample with stratification targets based on two criteria: geographic region and settlement size. Croatia has approximately 1200 community pharmacies (excluding hospital pharmacies), and stratification targets were set to reflect the distribution across four major geographic regions (Zagreb area, Adriatic coast, Northern Croatia, and Slavonia/Eastern Croatia) and five settlement size categories (regional centers over 100,000 residents, large cities 50,000–100,000, medium cities 10,000–50,000, small towns under 10,000, and rural areas). However, within strata, participation was based on voluntary self-selection; the sample is therefore not strictly probabilistic and should not be considered nationally representative. Pharmacists were invited to participate through the professional network Croatian Pharmacists Chamber and participation was voluntary. A total of 184 community pharmacies were invited to participate in the study. Recruitment was conducted primarily via email, with telephone follow-up introduced during the later phase of data collection to support participation. Participation was voluntary and based on self-selection. Of the invited pharmacies, 100 completed the survey, yielding a participation rate of 54% (100/184). For pharmacies that did not respond or declined to participate, other pharmacies sharing similar region and settlement size characteristics were substituted and invited. One pharmacist per pharmacy participated in the study to ensure independence of observations. Information on professional role, years of experience, and pharmacy characteristics (ownership and chain affiliation) was collected to describe the sample. Within each pharmacy, we documented whether the respondent was the pharmacy owner, Master of Pharmacy, or pharmaceutical technician. We also collected information about pharmacy type (state/city-owned versus private) and chain affiliation (independent versus small or large chain) to identify potential systematic differences in the pharmacy sample. Participating pharmacists had substantial professional experience and spent an average of 83% of their work time in direct patient contact (Table 1). The majority (73%) held Master of Pharmacy degrees, with the remainder being pharmaceutical technicians working at the dispensing counter. The achieved distribution across strata is presented in Table 2; given the voluntary nature of participation and underrepresentation of certain settlement size categories, findings should be interpreted with appropriate caution and should not be generalized to all Croatian community pharmacies.

### 2.3. Patient Record Form (PRF) Data Collection

Each pharmacist prospectively collected PRFs over a four-week period during September–October 2025. The protocol specified that pharmacists complete a brief PRF immediately following every patient encounter involving an ONS prescription requiring co-payment. This real-time documentation approach was critical for accuracy and completeness.

Each PRF captured the following: patient demographics (age, gender, retirement status), primary diagnosis necessitating ONSs, presence of comorbidities and co-payment for other medications, prescribed ONS brand and dosage (number of bottles/packages), whether this was the patient’s first ONS prescription or a refill, pharmacist’s assessment of whether the patient knew in advance about co-payment requirements, patient’s immediate response to learning the co-payment amount (accepted immediately, requested additional explanation then accepted, requested cheaper alternative or smaller dose, or refused entirely), and, if refused, the pharmacist’s professional assessment of the primary reason. These assessments were based on the pharmacist’s interpretation of the patient interaction at the time of dispensing.

Each pharmacist was asked to complete approximately 13 PRFs during the four-week collection period. Some pharmacists completed fewer forms due to lower patient volumes, so additional pharmacists were recruited to reach the target. Ultimately, 973 fully completed PRFs were analyzed.

The brief duration of each PRF (1–2 min) and the easily recollectable nature of the data (information just discussed with the patient), combined with financial incentives for participation, ensured high-quality data collection with minimal pharmacist burden. All patient data were collected anonymously with no personally identifying information recorded, and pharmacists provided informed consent acknowledging their voluntary participation and understanding of the research purpose.

### 2.4. Statistical Analysis

Data was analyzed using SPSS software (IBM SPSS Statistics for Windows, Version 24.0. Armonk, NY, USA: IBM Corp.). Descriptive statistics are used to describe the basic features of the sample in a study (proportions for categorical data and mean +/− standard deviation). Binary or categorical outcome variables were analyzed with chi-square test or Fisher’s exact test (alternative to the chi-square for 2 × 2 contingency tables). Univariate binary logistic regression analyses were performed to examine the association between each independent variable and the outcome (refusal of ONS co-payment). Variables that were statistically significant in univariate analyses were subsequently included in the multivariate logistic regression model with addition of diagnosis due to its clinical importance (binarized to oncological vs. other included diagnosis). Given the limited number of events, the multivariable analysis was conducted for exploratory purposes, with predictors selected a priori based on theoretical relevance. Variables with a *p*-value < 0.10 in univariate analyses were considered for inclusion in the multivariate logistic regression model.

## 3. Results

### 3.1. Study Population Characteristics

The final analyzed dataset comprised 973 patient encounters, with demographics shown in Table 3. The patient population reflected the expected demographics of individuals requiring ONS therapy in a European healthcare system.

**Figure 1:**
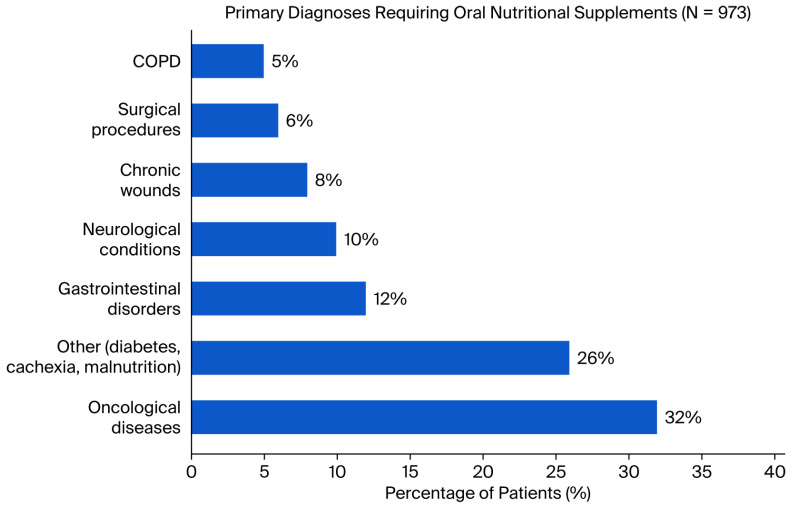
Primary diagnoses requiring ONSs (N = 973).

The diagnostic distribution (Figure 1) reveals substantial heterogeneity in the patient population, with oncological diseases representing the single largest category (32%). The large ‘other’ category (26%) predominantly comprises patients with diabetes-related malnutrition, cachexia without identified underlying causes, and general protein-energy malnutrition, reflecting the complex and often multifactorial nature of malnutrition in clinical practice.

### 3.2. Treatment Patterns and Prescribing Practices

Analysis of prescription patterns provided insight into real-world ONS prescribing practices in a co-payment system is shown in Table 4.

Patient preparedness for the pharmacy encounter varied notably by prior experience with ONS therapy. While 65% of all patients knew in advance about ONS co-payment requirements, 51% of first-time users arrived at the pharmacy uninformed or insufficiently informed about the associated costs. This knowledge gap proved consequential for acceptance patterns and the quality of the pharmacist–patient interaction, shown in Table 5. Patient responses to co-payment disclosure for all patients and first-time users are shown in Table 6.

### 3.3. Determinants of ONS Prescription Refusal

For the 3% of patients (N = 26) who refused their ONS prescription entirely, pharmacists recorded their assessment of the primary reason for refusal at the time of the encounter. These are pharmacist-assessed proxies based on observed patient behavior and interactions, not validated patient-reported outcomes. With this caveat in mind, the data suggest that cost, while predominant, was not the only barrier identified by pharmacists (Table 7).

The characteristics of patients who refused ONSs are shown in Table 8. While financial burden was cited in 59% of refusals, 23% of patients that refused prescribed ONSs appeared not to understand why ONSs were prescribed or what benefits to expect.

Pharmacists state that education about the benefits and necessity of ONSs in certain diagnoses would be most useful in their day-to-day work with patients (Table 9).

Univariate binary logistic regression analyses were performed to examine the association between each independent variable and the outcome (Table 10). Variables that were statistically significant in univariate analyses were subsequently included in the multivariate logistic regression model. Diagnosis was retained in the multivariable model due to its clinical relevance despite not reaching statistical significance. Variables with a *p*-value < 0.10 in univariate analyses were considered for inclusion in the multivariate logistic regression model.

The binary logistic regression model was then calculated to determine what factors significantly contribute to the criterium variable (refusal of ONS co-payment). The model explains a modest portion at 15% of variance of the criterion (Nagelkerke R2 = 0.149). The multivariable logistic regression model was statistically significant (Omnibus test: χ^2^(4) = 14.48, *p* = 0.006) and demonstrated good calibration (Hosmer–Lemeshow test *p* = 0.967).

The model reveals that only knowing about co-payment prior to coming to the pharmacy (OR = 0.115, *p* = 0.005, 95% CI: 0.025–0.528) is in fact a statistically significant predictor of co-payment ONS refusal, meaning that those patients who knew they needed to co-pay had 88.5% significantly lower odds of refusal that those who were not informed about co-payment.

## 4. Discussion

This study examined the ONS provision pathway in Croatia’s co-payment healthcare system and identified several communication and preparedness gaps that may be associated with reduced ONS initiation and may represent modifiable targets for future intervention. The predominantly elderly patient population (73% over age 60) aligns with epidemiological expectations for disease-related malnutrition, which disproportionately affects older adults due to age-related physiological changes, multiple chronic conditions, polypharmacy effects, and social factors including isolation and economic constraints [9,16].

The high prevalence of comorbidities (53% confirmed, with an additional 22% where pharmacists could not definitively assess) underscores the complex medical profiles typical of ONS users [17]. Among patients with documented comorbidities, cardiovascular disease was most prevalent (66%), followed by diabetes (24%) and mental health disorders (21%). This comorbidity pattern has important implications for patient capacity to manage multiple treatment regimens and cumulative co-payment burdens.

Notably, 65% of patients had other co-paid medications, meaning the ONS co-payment represented an additional financial burden atop existing medication costs [18], with this cumulative cost consideration proving relevant for understanding patient acceptance patterns.

The observed distribution of bottle counts is notable in the context of standard ONS dosing guidance—prescription of two bottles/bags a day as a recommended dose due to the content of energy, protein, or pharmaconutrients [19]. However, interpretation of this distribution as under-prescribing or appropriate prescribing is not possible without data on individual patient body weight, disease severity, functional status, or prescriber intent. Several factors might explain this pattern: prescriber caution about patient acceptance of co-payment costs, uncertainty about patient tolerance or adherence during initial treatment phases, insurance or reimbursement policies that may limit prescription quantities, or clinical decision-making that favors shorter prescriptions for reassessment [20].

The 92% overall acceptance rate of prescribed ONSs (77% immediate acceptance plus 15% after pharmacist explanation) indicates that most patients successfully obtained their prescribed ONSs. However, 8% experienced significant barriers (3% refusing entirely, 5% requesting cheaper alternatives or reduced doses), representing nearly 1 in 12 patients who did not receive therapy as prescribed. First-time ONS users showed a higher rate of non-acceptance (12% with acceptance barriers versus 8% in the overall cohort), suggesting that the first prescription encounter may be a particularly relevant point in the care pathway where communication and preparedness gaps are associated with non-initiation of medical nutrition therapy.

While 65% of all patients knew about co-payment requirements in advance, 51% of first-time users arrived without adequate prior knowledge of co-payment requirements. This knowledge gap had notable consequences for acceptance outcomes: patients who arrived informed showed 93% immediate acceptance with only 3% requiring additional explanation, whereas uninformed patients showed only 33% immediate acceptance and 49% required pharmacist explanation before deciding (*p* < 0.05).

This marked divergence in acceptance patterns based on prior knowledge suggests a potential gap in prescriber–patient communication at the point of prescription. Collaborative practice between healthcare providers has been associated with better-quality care and health outcomes for patients, while gaps in interprofessional communication have been linked to patient dissatisfaction, medication errors, and adverse outcomes [21]. Prior awareness of co-payment was strongly associated with immediate acceptance, suggesting that informational preparedness may play an important role alongside financial barriers.

Among the 26 patients who refused ONSs, pharmacist-assessed barriers extended beyond financial constraints. While 59% cited expensive co-payment as the primary reason, 23% appeared not to understand the prescribing rationale or expected benefits—a finding that may suggest insufficient therapeutic communication at the point of prescription, though it is based on pharmacist assessment of a small subgroup and should be interpreted cautiously. Pharmacists identified education regarding ONS benefits and clinical indication for specific diagnoses as their primary unmet educational need (Table 9), which is consistent with reports that ONS-specific training in pharmacy curricula is often limited [22].

In univariate logistic regression analyses, three variables (knowing about co-payment prior to coming to the pharmacy, frequency of collecting ONSs (first time or second and other times), and co-payment of other therapy) were associated with the outcome and were therefore considered for inclusion in the multivariable model, based on statistical association and clinical relevance. Given the limited number of outcome events (N = 26), the multivariable model should be interpreted as exploratory and hypothesis-generating rather than confirmatory. With this caveat in mind, only one variable showed statistically significant associations with co-payment refusal in the multivariable model: knowing about co-payment prior to coming to the pharmacy (OR = 0.1157, 95%CI: 0.025–0.528, *p* = 0.005), suggesting that patients who knew they needed to co-pay had lower odds of refusal than those who were not informed. These estimates should be interpreted with caution given the potential for sparse-data bias and model instability. The observed association between prior knowledge and reduced refusal (97% reduced odds of refusal) is consistent with research reporting associations between effective clinician–patient communication about treatment costs and expectations and improved adherence [23]. This observation is also consistent with the shared decision-making framework, in which transparent communication about financial obligations has been linked to greater patient preparedness and treatment engagement [24].

In univariate analysis, patients already managing co-payments for other therapies showed lower odds of refusing ONSs (OR = 0.250, *p* = 0.002). However, this association did not retain statistical significance in the multivariable model (OR = 0.649, *p* = 0.500), suggesting that the apparent protective effect of co-payment experience may be explained by other variables included in the model, most plausibly prior knowledge of ONS co-payment requirements. This pattern is consistent with research indicating that patients managing multiple therapies may develop broader coping strategies for healthcare costs [25,26], but the loss of significance in the adjusted model precludes any firm conclusion.

### 4.1. Practical Implications

The present findings identify several gaps in the ONS care pathway that may warrant targeted attention in clinical practice and future intervention research. The following implications are derived from the study data and should be understood as hypothesis-generating rather than evidence-established recommendations, given the observational and cross-sectional nature of this study [24,27,28].

Three areas of the care pathway appear relevant based on the observed findings:

Prescriber–Patient Communication: The findings that 51% of first-time users arrived without prior knowledge of co-payment requirements and that uninformed patients showed markedly lower immediate acceptance (33% versus 93%) suggest that explicit prescriber communication about co-payment obligations and therapeutic rationale may be associated with improved uptake. Whether structured prescriber–patient communication at the point of prescription would reduce refusal rates in this population warrants prospective evaluation [23].

Prescriber–Pharmacist Communication: The observations that 15–22% of patients required pharmacist explanation before accepting their prescription and that 23% of those who refused appeared not to understand the therapeutic rationale point to a potential gap in prescriber–pharmacist information transfer. Structured bidirectional communication between prescribers and pharmacists—covering clinical rationale, patient preparation, and feedback on acceptance—may be worth examining as a modifiable factor, consistent with evidence linking interprofessional communication with care quality [29].

Pharmacist Education: Pharmacists in this study identified disease-specific nutritional knowledge as their primary unmet educational need (Table 8), and a substantial proportion of patients required pharmacist intervention before accepting their prescription. These observations suggest that targeted continuing professional development in clinical nutrition and ONS counseling may support pharmacists in this role. Whether such training translates into improved patient acceptance or adherence remains to be tested in future intervention studies [22].

### 4.2. Limitations

Several limitations of this study warrant consideration. First, the sampling approach introduces potential for selection bias. The sample was designed as a structured pragmatic sample with stratification targets based on geographic region and settlement size; however, participation within strata was voluntary and based on self-selection, and the sample is therefore not strictly probabilistic. Pharmacies or pharmacists with a particular interest in nutrition, patient counseling, or professional development may have been more inclined to participate, which could mean that the observed acceptance rates and pharmacist engagement levels are higher than would be found across the broader Croatian community pharmacy population. The 54% participation rate (100 of 184 invited pharmacies) means that approximately half of contacted pharmacies did not contribute data; characteristics of non-participating pharmacies are unknown. Accordingly, findings should not be interpreted as nationally representative and should be treated as reflecting the experience of a structured pragmatic sample approximating the national distribution by region and settlement size. Second, the cross-sectional observational design captures a single point of interaction and does not facilitate follow-up on patient adherence or long-term outcomes. Acceptance at the dispensing counter does not guarantee sustained use, and the magnitude of actual treatment non-adherence over time may differ from the acceptance barriers documented here.

Third, several key variables—including patients’ prior knowledge of co-payment requirements, reasons for refusal, and patient understanding of therapeutic rationale—were recorded based on the pharmacist’s assessment of the encounter rather than direct patient self-report. These are pharmacist-assessed proxies, not validated patient-reported outcomes, and do not necessarily reflect the patient’s own perspective or experience. Although real-time documentation at the point of dispensing reduces recall bias, the data may nonetheless reflect the pharmacist’s interpretation of patient behavior, introducing the possibility of observer and interpretation bias. Future studies should consider incorporating direct patient-report instruments alongside pharmacist assessment to allow comparison and validation of these proxy measures. Fourth, the study was conducted over a four-week period in a single country with a specific co-payment model, limiting external validity to other healthcare systems, reimbursement structures, or seasonal patterns in prescribing ONSs. Finally, while the sample size of 973 patient encounters is adequate for the primary analyses, the number of patients who refused ONSs entirely (N = 26) is small, and subgroup analyses within this group should be interpreted cautiously. The relatively small number of outcome events (N = 26) in relation to the number of predictors included in the multivariable model may increase the risk of overfitting and model instability; therefore, the regression findings should be interpreted as exploratory.

Fifth, although the primary unit of analysis was the individual patient encounter, observations are partially clustered within pharmacies, with each pharmacy contributing approximately ten records. Clustering by pharmacy was not explicitly modeled in the statistical analysis; given the small and relatively balanced number of observations per site, the impact of intraclass correlation was considered likely limited. Nevertheless, responses from patients within the same pharmacy may not be fully independent, and future studies with larger within-pharmacy samples should consider multilevel or cluster-robust analytical approaches.

## 5. Conclusions

This study examined the ONS provision pathway in a co-payment healthcare system and identified several factors associated with acceptance and refusal at the pharmacy dispensing encounter. Prior knowledge of co-payment requirements showed the strongest association with acceptance in the exploratory regression model, with informed patients showing markedly lower odds of refusal compared to uninformed patients. Overall, 8–12% of patients did not collect their prescribed ONSs as dispensed; financial barriers and insufficient therapeutic understanding were the most frequently pharmacist-assessed barriers among those who refused. These findings suggest that the pharmacy dispensing encounter is a clinically relevant point in the ONS care pathway, where gaps in prescriber–patient and prescriber–pharmacist communication may be associated with suboptimal treatment uptake. Enhanced collaborative practice across prescribers, pharmacists, and patients—including systematic co-payment counseling, disease-specific therapeutic education, and structured interprofessional communication—may help reduce barriers to ONS initiation in similar healthcare settings. Further prospective studies are needed to evaluate whether targeted interventions at the dispensing encounter improve long-term adherence and nutritional outcomes in patients with disease-related malnutrition.

## Figures and Tables

**Table 1:** Pharmacist sample characteristics.

Characteristic	Value
Total pharmacists	N = 100
Average length of work experience	16 years (SD = 9, range 1–37)
Direct patient contact time	83% of work time
Education level	73% Masters of Pharmacy

**Table 2:** Comparison of the sample distribution to the national sample.

	Sample	Population	Deviation
Region			
Zagreb	55%	48%	7%
Osijek	11%	17%	−6%
Rijeka	14%	14%	0%
Split	20%	21%	−1%
Settlement Size			
Regional center (over 100,000 residents)	34%	30%	4%
Large cities (50,000–100,000)	9%	19%	−10%
Medium-sized town (10,000–50,000)	14%	10%	4%
Small town (under 10,000) and rural area	43%	41%	2%

**Table 3:** Patient demographics (N = 973).

Characteristic	Distribution
Gender	51% female, 49% male
Mean age	68 years (SD = 15.4, range 12–96)
Age ≥ 60 years	73%
Comorbidities present	53% confirmed, 22% uncertain
Other co-paid therapy	65%

**Table 4:** Prescribed dose of oral nutritional supplements (N = 973).

Prescribed Dose	Percentage
Up to 10 bottles/bags	14%
11 to 20 bottles/bags	17%
21 to 30 bottles/bags	32%
31 to 40 bottles/bags	12%
41 to 50 bottles/bags	2%
51 to 60 bottles/bags	20%
More than 60 bottles/bags	2%
Up to 30 bottles/bags (≤1 month supply)	63%

**Table 5:** Patient awareness of co-payment requirements and impact on acceptance.

	Aware	Unaware
Patient Group		
All patients	65%	35%
First-time ONS users	49%	51%
Impact on acceptance		
immediate acceptance	93%	33%
needed explanation	3%	49%
refused	1%	9%

**Table 6:** Patient response depending on category—all patients vs. first-time users (N = 973).

Response Category	All Patients	First-Time Users
Accepted immediately	77%	64%
Accepted after explanation	15%	22%
Total accepted full prescription	92%	86%
Requested cheaper/less	5%	7%
Refused entirely	3%	5%
Total with acceptance barriers	8%	12%

**Table 7:** Primary reasons for ONS refusal (N = 26).

Reason for Refusal	Percentage
Co-payment too expensive	59%
Does not understand benefit/necessity	23%
Poor mental state	8%
Non-adherence to therapy	8%
Other reasons	2%

**Table 8:** Characteristics of patients who refused ONSs (N = 26).

Characteristic	Finding
Gender	54% female, 46% male
Mean age	64 years (range 35–84)
Retired	50%
Had comorbidities	38% (mainly cardiovascular)
Other co-paid therapy	35%
Knew about co-payment	31% (69% unaware)
First-time users	50%
Geographic pattern	Higher in Osijek region
Settlement size	Higher in large cities (50–100 K)

**Table 9:** Pharmacist-identified educational needs.

Support Needed	Priority
Education about ONS benefits	Highest priority
Disease-specific nutritional needs	High priority
When ONSs are medically necessary	High priority
Expected patient outcomes	Moderate priority
Product differentiation	Lower priority

**Table 10:** Univariate binary logistic regression indicators, and binary logistic regression model with only significant predictors included (statistically significant *p*-values are highlighted in bold).

Variables	Univariate Binary Logistic Regressions			Binary Logistic Regression Model		
	OR	*p*	95%CI	OR	*p*	95%CI
Diagnosis (oncological)	0.706	0.497	0.258–1.930	0.302	0.132	0.064–1.433
Diagnosis (neurological, GI, COPD, surgical, chronic wounds)—ref						
Age (up to 60 yrs)	1.623	0.384	0.546–4.829			
Age (61 to 80 yrs)	0.840	0.753	0.283–2.489			
Age (81+ yrs)—ref						
Knew about co-payment	0.062	**<0.000**	0.021–0.186	0.115	**0.005**	0.025–0.528
Did not know—ref						
Frequency of collecting ONSs (first time)	3.732	**0.002**	1.612–8.639	0.737	0.673	0.179–3.038
Second and other times—ref						
Co-payment of other therapy	0.250	**0.002**	0.104–0.601	0.649	0.500	0.184–2.291
No co-payment—ref						
Region (Zagreb and North Croatia)	0.984	0.977	0.342–2.831			
Region (Slavonia)	2.238	0.192	0.667–7.510			
Region (Istria and north Kvarner)	0.939	0.932	0.220–3.998			
Region (Middle and South Dalmatia)—ref						
Settlement size (regional center)	0.808	0.708	0.266–2.460			
Settlement size (large city)	1.684	0.422	0.472–6.007			
Settlement size (medium-sized city)	0.624	0.525	0.146–2.669			
Settlement size (small town)	0.378	0.152	0.100–1.431			
Settlement size (rural area)—ref						

## Data Availability

The raw data supporting the conclusions of this article will be made available by the authors on request.

## Abbreviations

The following abbreviations are used in this manuscript:

ONS	Oral Nutritional Supplement
DRM	Disease-Related Malnutrition
FSMP	Food For Special Medical Purposes
NST	Nutrition Support Therapy
PRF	Patient Record Form
